# Adaptation of Water, Sanitation, and Hygiene Interventions: A Model and Scoping Review of Key Concepts and Tools

**DOI:** 10.3389/frhs.2022.896234

**Published:** 2022-05-09

**Authors:** Darcy M. Anderson, Sarah A. Birken, Jamie K. Bartram, Matthew C. Freeman

**Affiliations:** ^1^Public Health and Environment, The Water Institute, Gillings School of Global Public Health, University of North Carolina at Chapel Hill, Chapel Hill, NC, United States; ^2^Department of Implementation Science, Wake Forest School of Medicine, Winston-Salem, NC, United States; ^3^Wake Forest Baptist Comprehensive Cancer Center, Medical Center Boulevard, Winston-Salem, NC, United States; ^4^School of Civil Engineering, University of Leeds, Leeds, United Kingdom; ^5^Gangarosa Department of Environmental Health, Rollins School of Public Health, Emory University, Atlanta, GA, United States

**Keywords:** adaptation model, evidence-based intervention (EBI), implementation science, water, sanitation, hygiene, WaSH

## Abstract

**Background:**

Safe water, sanitation, and hygiene (WaSH) is important for health, livelihoods, and economic development, but WaSH programs have often underdelivered on expected health benefits. Underperformance has been attributed partly to poor ability to retain effectiveness following adaptation to facilitate WaSH programs' implementation in diverse contexts. Adaptation of WaSH interventions is common but often not done systematically, leading to poor outcomes. Models and frameworks from the adaptation literature have potential to improve WaSH adaptation to facilitate implementation and retain effectiveness. However, these models and frameworks were designed in a healthcare context, and WaSH interventions are typically implemented outside traditional health system channels. The purpose of our work was to develop an adaptation model tailored specifically to the context of WaSH interventions.

**Methods:**

We conducted a scoping review to identify key adaptation steps and identify tools to support systematic adaptation. To identify relevant literature, we conducted a citation search based on three recently published reviews on adaptation. We also conducted a systematic database search for examples of WaSH adaptation. We developed a preliminary model based on steps commonly identified across models in adaptation literature, and then tailored the model to the WaSH context using studies yielded by our systematic search. We compiled a list of tools to support systematic data collection and decision-making throughout adaptation from all included studies.

**Results and Conclusions:**

Our model presents adaptation steps in five phases: intervention selection, assessment, preparation, implementation, and sustainment. Phases for assessment through sustainment are depicted as iterative, reflecting that once an intervention is selected, adaptation is a continual process. Our model reflects the specific context of WaSH by including steps to engage non-health and lay implementers and to build consensus among diverse stakeholders with potentially competing priorities. We build on prior adaptation literature by compiling tools to support systematic data collection and decision-making, and we describe how they can be used throughout adaptation steps. Our model is intended to improve program outcomes by systematizing adaptation processes and provides an example of how systematic adaptation can occur for interventions with health goals but that are implemented outside conventional health system channels.

## Introduction

Despite decades of effort and investment, water, sanitation, and hygiene (WaSH) interventions in low- and middle-income countries have often failed to deliver expected health benefits, and even infrastructure access and behavioral outcomes have been poorly sustained. WaSH interventions have shown health benefits in small-scale pilots and tightly controlled trial settings. However, adapting these evidence-based interventions to facilitate implementation at-scale in ways that maintain originally demonstrated levels of effectiveness has proved challenging (1–3). These challenges have been attributed in part to poor ability to adapt to context and retain effectiveness as interventions are implemented at scale (4–10).

Rapid scale-up has outpaced learning on what adaptations are necessary and effective processes for making them. Across WaSH interventions, adaptation is common but often does not follow a systematic process (11, 12). Overall, there is little guidance on how adaptation should occur throughout WaSH intervention development, implementation, and sustainment. In some cases, documentation of adaptation may be actively suppressed, as implementers sometimes perceive that any modification to the original intervention is undesirable (11).

Adaptation literature as a subfield of implementation science provides concepts and tools that could be leveraged to improve adaptation in the WaSH sector. The past decade has seen substantial advancement of theoretical tools and methods to understand how, when, and why to adapt, and to support systematic and replicable study of adaptations and their effects on program outcomes (13–17). Theoretical tools and systematic methods from adaptation literature have been successfully applied for improving outcomes in health programming, but, to the best of our knowledge, have yet to be applied in WaSH.

Most published literature of WaSH intervention adaptation reports adaptation in the early stages of programming, typically in intervention design stage before any implementation has been done, or in the pilot stage before implementation at-scale [see, e.g., (18–21)]. These studies report adaptations designed and implemented with strong support from academics. This poorly reflects the realities of WaSH programs, where adaptation is an ongoing process done by practitioners throughout implementation and rarely applies academic frameworks and theories (12).

We argue that adaptation models and frameworks have been underused in WaSH to date but offer valuable insights for achieving effectively implementing and sustaining WaSH interventions. These tools, when applied in the health sector, have been shown to improve outcomes such as intervention acceptability, adoption, and sustainability (16, 22). However, existing adaptation models were predominantly developed in the context of healthcare delivery [see, e.g., (17, 23)]. While WaSH interventions have health goals, they also have non-health human rights and development goals. Implementation and regulation are often divided across different ministries and interest groups. At the local level, some implementers may be health workers (e.g., community health workers), but overall stakeholders are diverse and include a variety of non-health and for-profit implementers (e.g., rural development committees, engineers, and small business owners). Existing adaptation models assume interventions are implemented by trained healthcare professionals, whereas in WaSH implementation by volunteers, beneficiaries, and laypersons is common. These contextual differences warrant more in-depth examination and tailoring of existing adaptation tools to better match the context of WaSH and similar interventions implemented outside traditional health system channels.

We developed a model specifically tailored to WaSH interventions to guide stakeholders through systematic adaptation. The purpose of this model is to improve adaptation by describing a series of key steps and providing tools to support systematic decision-making throughout, thereby improving outcomes. We propose that applying this model will improve adaptation in WaSH by systematizing the adaptation process and reducing unsystematic decision-making (12, 24).

## Methods

We followed the five steps for scoping reviews outlined by Arksey and O'Malley (25): (1) identifying the research question, (2) identifying relevant studies, (3) selecting studies, (4) charting the data, and (5) collating, summarizing, and reporting the results. We conducted a scoping review as opposed to a systematic review to explore the breadth of available adaptation literature and capture evolving concepts and terminology that would be difficult to capture through a systematic review approach (25, 26).

### Identifying the Research Question

Our scoping review was guided by two research questions: (a) What are the steps required for systematic intervention adaptation in WaSH? and (b) What tools exist to guide these adaptation steps?

We focused our review on WaSH interventions targeting drinking water, sanitation, and handwashing with soap. We included adaptations to interventions that were user-facing (e.g., household toilets and sinks) and non-user facing (e.g., municipal water and sewage treatment plants). We considered the following to be out of scope: water resource management for recreational, agricultural, and other non-consumption uses; non-sewerage wastewater treatment; and hygiene programs without a handwashing component.

### Identifying Relevant Studies

#### Scoping Search

We began with three recent reviews that have synthesized models of key adaptation steps from prior literature: Movsisyan et al. (15), Kirk et al. (16), and Escoffery et al. (17). We then searched for updates, refinements, or alternatives to these models using forward and backward citation searching in Google Scholar. Forward and backward citation searching entails beginning with an initial set of seed studies—in our case reviews by Movsisyan, Kirk, and Escoffery—then reviewing the references cited therein and references that have subsequently cited those seed studies. This approach has been found to have comparable success in identifying relevant papers as keyword searches, and is recommended for exploratory, scoping reviews where an initial narrowly defined keyword search may not capture all relevant concepts (27).

To identify tools to guide the adaptation process, we identified three additional recent reviews of adaptation theory and practice (13, 14, 28) and repeated our process of citation searching. We also reviewed three recent papers on implementation science in WaSH and environmental health (1, 29, 30).

#### Systematic Database Search

We conducted a systematic search to identify references describing adaptations in WaSH. We searched PubMed, Web of Science, and Scopus for English language studies published between 1^st^ January 2000 and 21 June 2021 (the search date). For a full listing of search terms, see Supplementary File 1.

### Study Selection

For studies identified through forward and backward citation searching in our scoping search, we reviewed the full text of identified studies and included those intended to be applicable to a wide variety of interventions. We excluded studies applicable only to a specific type of intervention or context not relevant to WaSH (e.g., models specific to HIV/AIDS programs). We prioritized publications from the past 5 years and stopped citation searching when we reached concept saturation (i.e., references did not yield new information or meaningfully refine our findings from previously identified references).

For studies identified through our systematic database search, we included studies that described adaptations to WaSH interventions delivered in households, institutional settings (e.g., healthcare facilities, schools), and water and sanitation utilities. We included retrospective studies of previous adaptations and prospective studies describing designing and planning adaptations for future implementation. We also included studies that presented tools to support adaptation. We excluded studies that identified a need for adaptation but undertook no further steps to design or implement any modifications. We assessed these inclusion and exclusion criteria in two rounds of screening: first of titles and abstracts, then the full-texts of studies that passed the initial screening.

### Charting the Data; Collating, Summarizing, and Reporting Results

First, we identified the adaptation steps described in the three adaption models that formed the basis of our literature search (15–17). We synthesized steps that were common across all models into a preliminary model of key steps. We also added steps that were not included in all models but reflected a new development in adaptation theory. We then compiled a list of actions required to complete each step.

Second, we conducted two rounds of revision to this preliminary model. We revised model steps based on updates or refinements from adaptation literature identified through forward and backward citation searching. We then revised our model based on the specific characteristics of WaSH programs. For studies of WaSH adaptations identified in our systematic database search, we documented the following: steps taken, stakeholders engaged, and barriers and facilitators. We used this information to tailor our model to WaSH interventions.

Third, we compiled a list of tools available to support data collection and decision-making for each step. We compiled these tools from both non-WaSH adaptation literature and WaSH-specific adaptation examples. We sorted these tools into groups based on their primary purpose (e.g., assessing needs, evaluating outcomes).

Our results summarize the scope of literature reviewed, review key adaptation principles based on included literature, and present our refined model of adaptation steps and the tools available to support each step. We then discuss how this paper synthesizes prior models and contributes to existing adaptation literature and the specific considerations for adaptation in a WaSH context.

## Results

### Scope of Literature Reviewed

Our model is grounded in previous adaptation models proposed by Movsisyan (15), Kirk (16), and Escoffery (17). We identified an additional 19 references in adaptation literature to refine and update our model (1, 10, 13, 14, 23, 24, 28–40). Our systematic search for WaSH adaptation studies yielded 1,649 unique references across all databases. After screening, we identified 30 relevant studies (18–20, 41–67) (Table 1). An overview of the setting, target population, and intervention of each of these 30 studies is included in Supplementary File 2.

**Table 1:** Studies of WaSH intervention adaptation yielded through systematic literature search.

**Intervention type**	**Number of studies**	
	**Low- and middle-income countries**	**High-income countries**
Climate change adaptation	6 (57–62)	4 (52–55)
Municipal utilities or communal water sources	1 (56)	2 (50, 51)
Rural household WaSH programs	7 (18, 20, 46–49, 63)	-
Urban household WaSH	5 (19, 42–45)	-
Hand hygiene in healthcare settings	2 (65, 67)	-
Schools	1 (66)	-
Integrating mobile monitoring technology	1 (41)	-
Public washrooms	-	1 (64)

### Key Principles in Adaptation

A key principle within adaptation literature is the distinction between core functions of an intervention (i.e., its purpose and mechanisms by which outcomes and health impacts are achieved) vs. its forms (i.e., the specific steps or activities used to carry out each core function) (40). The purpose of adaptation is to alter the forms of an intervention to improve performance, while preserving the underlying core functions. Distinguishing between core functions vs. forms allows for flexibility to deviate from the original specific activities of an intervention, while retaining the underlying mechanisms that drives its effectiveness.

Where adaptations do not appropriately identify and preserve core functions, they risk sacrificing effectiveness (16, 36, 38, 40). For example, if core functions are not well understood, adaptations may unknowingly undermine them, resulting in no improvement or even worse performance (11). Stakeholders can iterate adaptations through trial and error, but this can be highly inefficient when numerous adaptations must be tried and discarded before a suitable adaptation is identified. Understanding core functions can improve efficiency by guiding selection of adaptations that are more likely to be successful from the start (68).

In some cases, particularly in WaSH interventions where core functions were not well-defined during intervention development, it may be appropriate to intentionally modify core functions. However, this should be done with good understanding of core functions of the original intervention, such that modifications are intentionally designed to improve them based on robust understanding of needs and context, rather than undermining them accidentally.

Terminology to describe key constructs and concepts within adaptation literature sometimes lacks consensus. To the extent possible, we have attempted to use the terms that best represent current consensus. For clarity and transparency, definitions of key terms as operationalized in this paper are presented in Table 2.

**Table 2:** Definitions of key terms as applied in this paper.

**Key terms**	**Definition**
Adaptations	Modifications made to interventions or implementation strategies to improve their performance (14, 15)
Interventions	The practices, products, policies, and procedures designed to improve WaSH conditions (e.g., installation of toilets and behavior change campaigns to promote their use) (69)
Implementation strategies	The methods or techniques used to improve the delivery, adoption, and sustainability of those interventions (70)
Intervention core functions	The purpose and mechanisms driving intervention impacts (i.e., how and why an intervention achieves change) (22)
Intervention forms	The specific products and activities used to achieve core functions of an intervention (22)

### A Model for Adapting WaSH Programs

#### Model Overview and Application

**Figure 1:**
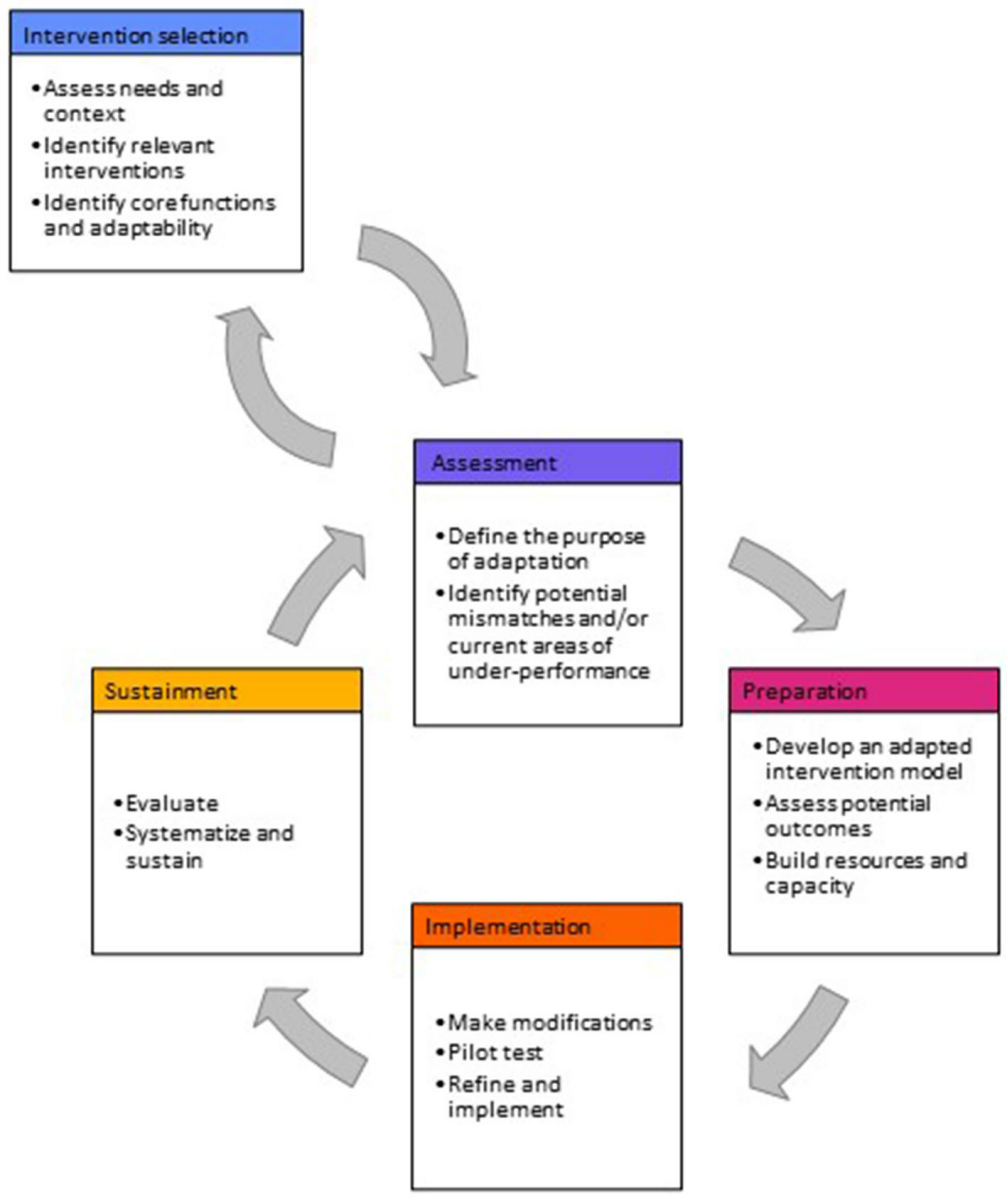
Steps for adaptation in WaSH programs. Model is informed by adaptation models by Movsisyan et al. (15), Kirk et al. (16), and Escoffery et al. (17) and refined based on case studies of adaptation in WaSH (18–20, 41–67).

Our model presents adaptation steps in five phases: intervention selection, assessment, preparation, implementation, and sustainment. Phases 2–4 (i.e., assessment through sustainment) are depicted as cyclical, reflecting the fact that once an intervention is selected, adaptation is an ongoing process that can be iterated to address unanticipated challenges and continually improve performance (16, 38). Each phase contains between two and four individual steps (Figure 1). Table 3 describes the specific actions required to complete each step. Table 4 presents tools that can support data collection and decision-making throughout the model steps.

**Table 3:** Description of actions for each adaptation step.

**Step**	**Key actions**	**Applicable tools**
***Intervention selection*** Stakeholders begin here when there is no existing intervention, and they are either (a) seeking a new intervention to adapt to meet a need, or (b) evaluating the suitability of a proposed intervention for the target population and its adaptation needs		
Assess needs and context	Conduct a needs assessment with the target population; identify vulnerable and marginalized groups and their specific needs Assess the context, including relevant facilitators and barriers to implementation, key stakeholders, and existing systems and support for implementation	Behavior change theories Bottleneck analysis Determinant frameworks Problem trees Stakeholder mapping
Identify relevant interventions	Identify salient intervention pathways and possible evidence-based interventions along those pathways Assess the fit of possible interventions to the target population Assess the fit of possible interventions to the context Evaluate evidence of effectiveness, population fit, and contextual fit; weigh possible trade-offs between outcomes; and select the best matching intervention	Evidence reviews and compendia Evaluation frameworks Multiple criteria decision analysis
Identify core functions and adaptability	Obtain the original intervention materials, such as logic models, stated goals and theory of change, curricula, training guides, etc. Identify the intervention's core functions Assess compatibility of core functions with the target population and context Assess the extent to which intervention forms can feasibly be adapted to the target population and context	Behavior change theories Intervention mapping Logic models
***Assessment*** Stakeholders adapting an existing intervention may skip the intervention selection phase and begin here. Before beginning the assessment phase, stakeholders should verify that steps to assess needs and context and to identify core functions and adaptability have already been completed during previous implementation efforts, or return to complete these actions as necessary to before beginning this phase.		
Define the adaptation purpose	Define the goals and purpose of adaptation If adapting an intervention for use in a new context, identify mismatches where intervention forms are a poor fit with the target population and context If adapting an existing intervention, describe current performance, desired performance, and performance gaps	Bottleneck analysis Determinant frameworks Multiple criteria decision analysis Problem trees
Determine adaptation needs	Describe the adaptations needed to fulfill the adaptation purpose Identify barriers and facilitators to adoption, implementation, and sustainability	Adaptation characteristics frameworks Bottleneck analysis Determinant frameworks
***Preparation***		
Develop an adaptation plan	Develop a logic model for the adapted intervention Develop a workplan for adapting and implementing the intervention Identify potential implementation strategies, leveraging existing infrastructure and resources, stakeholder capacities, etc. identified in the contextual assessment	Evidence reviews and compendia Logic models
Develop the adapted intervention package	Modify the intervention forms (e.g., products, policies, activities) through collaborative efforts with implementing stakeholders and communities Solicit and incorporate feedback from vulnerable and marginalized groups to tailor adapted intervention components as needed Review core functions and ensure they are retained in the adapted forms Develop an adapted intervention package, including activities guides, implementation manuals, training materials, monitoring and evaluation plan, dissemination plan	Behavior change theories Evaluation frameworks Intervention mapping Logic models
Build stakeholder support, resources, and capacity	Secure necessary formal and informal support from regulatory agencies (e.g., local or regional government, community management committees) Solicit feedback on the adapted intervention package from implementing partners and communities; refine as needed based on feedback Build support among implementing stakeholders for the adapted intervention package; identify champions among lay implementers from the target population Assess stakeholders' implementation capacity and readiness for change; assign roles and responsibilities Recruit and train implementation personnel, including personnel representative of and familiar with the target population and context Ensure vulnerable and marginalized groups are appropriately represented and engaged; ensure intervention and implementation plan meet community needs and values	Determinant frameworks Evaluation frameworks Stakeholder mapping
***Implementation***		
Pilot test	Pilot test the adapted intervention package with the target population Assess implementation outcomes (e.g., acceptability, adoption, appropriateness, fidelity to the adapted intervention plan, feasibility) Identify barriers and facilitators to implementing the adapted intervention Identify any unanticipated and/or unintended effects; revise as necessary to mitigate detrimental unintended effects Solicit and incorporate feedback from the target population, implementation personnel, and other stakeholders	Bottleneck analysis Determinant frameworks Evaluation frameworks Process evaluation
Refine and implement	Refine the intervention package based on pilot testing Establish systems for ongoing monitoring, refinement, and support for implementation Implement the adapted intervention	Multiple criteria decision analysis
***Sustainment***		
Evaluate	Monitor the implementation process following the adapted monitoring and evaluation plan Identify implementation challenges and provide support for quality implementation as needed Evaluate implementation outcomes and impacts of the adapted intervention Disseminate findings to stakeholders, solicit and incorporate feedback into the adapted intervention package	Bottleneck analysis Determinant frameworks Evaluation frameworks Process evaluation
Systematize and sustain	Sustain monitoring and evaluation to ensure satisfactory performance over time Identify opportunities for scale up, given satisfactory performance Identify opportunities to integrate and systematize the adapted intervention package into organizations, infrastructure, and other systems within the context Document and disseminate lessons learned throughout the adaption process	Bottleneck analysis Determinant frameworks Evaluation frameworks Process evaluation

*Adaptation steps are informed by adaptation models by Movsisyan et al. (15), Kirk et al. (16), and Escoffery et al. (17) and refined based on case studies of adaptation in WaSH (18–20, 41–67). Descriptions of the tools can be found in Table 4*.

**Table 4:** Tools to support adaptation.

**Tool**	**Function**	**Example(s)**	**Applicable model steps**
***Assessing context and conditions***			
Bottleneck analysis	Identifies and maps barriers to a specific process or to achieving a specific objective	Jimenez et al. (47) apply bottleneck analysis to identify barriers to sustainability for community-based water and sanitation services	Assess needs and context Define the adaptation purpose Determine adaptation needs Determine adaptation needs Pilot test Evaluate Systematize and sustain
Problem trees	Identifies proximal and distal causes of a community need or problem, maps relationships between causes, and identifies possible intervention pathways; can map intended and unintended downstream results	Tidwell et al. apply (19) participatory methods where community members are asked to rank causes of poor sanitation, then group related factors and identify possible intervention points	Assess needs and context Define the adaptation purpose
Stakeholder mapping	Describes different types of stakeholders and assesses their priorities, roles, and capacities in the adaptation process	Sigel et al. (44) identified all stakeholders directly or indirectly able to affect urban sanitation in target areas, and assessed their roles and responsibilities for sanitation delivery to ensure all relevant stakeholder were engaged in adaptation planning Medilanski et al. (42) mapped relevant stakeholder and their priorities to adapt the implementation plan and implementing partners for greater intervention uptake	Assess needs and context Build stakeholder support, resources, and capacity
***Understanding core functions and selecting adaptations***			
Behavior change theories	Describes drivers of behavior change and/or explains how and why behavior change occurs	Dawson et al. (64) apply the technology acceptance model (71), which predicts acceptance of new technologies based on perceptions of usefulness and ease of use, to assess the likely adoption of an adapted hand hygiene station	Assess needs and context Identify core functions and adaptability Develop the adapted intervention package
Intervention mapping	Applies a theory-driven process understanding community needs and causal factors, selecting theory-based intervention methods, and developing and implementing program components that can be used to understand core functions	Tidwell et al. (19) apply a theory-driven approach for developing an intervention to adapt and improve urban household toilets, first assessing community needs, then identifying theory-driven solutions to improve toilets.	Identify relevant interventions Identify core functions and adaptability Develop the adapted intervention package
Evidence reviews and compendia	Compiles evidence-based interventions and synthesizes available evidence of intervention effectiveness	International Initiative for Impact Evaluation's evidence map documenting intervention types and effectiveness at achieving various health and development outcomes (72) Systematic reviews and meta-analyses documenting impacts of WaSH interventions on health outcomes (73–79)	Identify relevant interventions Develop an adaptation plan
***Describing adaptations and implementation processes***			
Adaptation characteristics frameworks	Provide constructs to categorize and describe types of modifications and/or potential mediators and moderators to successful implementation	Stirman's Framework for Reporting Adaptations and Modifications to Evidence-based interventions (FRAME) (31) provides a typology for describing what aspect of programming was modified, by whom, when, and the extent to which adaptations are aligned with core functions	Determine adaptation needs
Determinant frameworks	Describe the factors that support and hinder implementation and sustainment of adaptations	Kohlit et al. (59) and Ojomo et al. (57) both propose framework of factors that support water systems in effectively adapting to climate change threats	Assess needs and context Define the adaptation purpose Determine adaptation needs Build stakeholder support, resources, and capacity Pilot test Evaluate Systematize and sustain
Logic models	Provides a graphical depiction of the inputs, outputs, outcomes, and impacts of a program, and the relationships between them	Movsisyan et al. (15) propose logic models as a tool to understand and describe core functions of adapted interventions	Identify core functions and adaptability Develop an adaptation plan Develop the adapted intervention package
***Evaluating adaptations and implementation processes***			
Evaluation frameworks	Presents constructs to assess adaptations' effects on program outcomes and impacts and/or provide guidance on the level of evidence needed to demonstrate that contextual fit has been improved while retaining core functions of an intervention and	Aarons et al. (10): define different types of scale up (e.g., new populations, new delivery systems, or both) and the level and types of new evidence that should be generated to demonstrate effectiveness under various scale-up scenarios Kirk et al. (16): propose evaluating adaptations' effects on eight implementation outcomes [acceptability, adoption, appropriateness, feasibility, fidelity, cost, penetration, sustainability (80)] to weigh potential trade-offs between intended and unintended effects	Identify relevant interventions Develop the adapted intervention package Build stakeholder support, resources, and capacity Pilot test Evaluate Systematize and sustain
Multiple criteria decision analysis	Provides a structured system of scoring and weighting various attributes to compare and rank different options	Anderson et al. (21) used a Pugh matrix (81) to score and weight the relative importance of acceptability, feasibility, and cost when choosing between prototypes of safe water storage containers	Identify relevant interventions Define the adaptation purpose Refine and implement
Process evaluation	Describes a systematic approach to assessing implementation quality and provides a framework for assessing relationships between contextual factors and implementation outcomes (e.g., dose delivered and received, program reach)	De Shay et al. (49) apply a process evaluation guide (82) to examine the effects of community perceptions on implementation of an adapted rural household sanitation intervention	Pilot test Evaluate Systematize and sustain

This model is designed to support two possible adaptation scenarios. In the first scenario, there is no pre-existing intervention. Stakeholders are selecting an intervention from another context to adapt for delivery in a new context. This may occur for several reasons. One, stakeholders have identified a health or development need that is not currently being met through an existing intervention. Stakeholders decide to meet this need through adapting an intervention from another context, rather than conducting *de novo* intervention development. Two, advocates for a particular intervention argue for its implementation in a new context, and adaptations are needed to improve contextual fit. In this case, stakeholders responsible for funding and implementing must determine if a need exists that warrants adapting and implementing this new intervention and whether other interventions would be more suitable. The first phase of the model (intervention selection) guides stakeholders through conducting a needs assessment, determining what interventions exist to meet this need, and selecting an intervention that is appropriate and adaptable in the target context. Subsequent phases of the model guide practitioners through adapting the selected intervention and implementing it in the new context.

In the second scenario, stakeholders have identified a need to adapt an existing intervention to improve its performance or address unanticipated problems encountered during implementation. In this case, stakeholders can skip the first phases of intervention selection and begin at the assessment phase. During assessment, stakeholders describe the purpose and specific needs for adaptation of the existing intervention, then proceed through subsequent phases of the model to prepare and implement the adaptation. In some cases, stakeholders in this scenario may still need to conduct selected actions under the intervention selection phase if they lack important information on an interventions' core functions or other contextual factors necessary for subsequent steps. In other cases, the assessment phase may reveal that an intervention is fundamentally incompatible with context, indicating a need to return to the intervention selection phase.

In both scenarios, this model is designed to guide stakeholders through a systematic adaptation process. Adaptations made through systematic processes have more beneficial effects on program outcomes, compared to adaptations that are unsystematic (12, 24). Steps in this model are intended to help stakeholders adhere to key principles of adaptation (i.e., identifying and preserving core functions), design effective adaptations, and understand adaptations' effects on program outcomes.

#### Adaptation Steps

In the intervention selection phase, stakeholders first assess the target population's needs and the context in which adaptation will occur. Stakeholders then identify possible interventions to address these needs and assess their core functions and adaptability (15, 17). This phase is intended for stakeholders who have identified a health need that is not currently met by an existing intervention and are adapting an intervention from another context. Stakeholders adapting an existing intervention may skip this phase.

In the assessment phase, stakeholders determine the purpose of the adaptation and identify adaptation needs. Where the purpose of adaptation is modifying an intervention for delivery in a new context, adaptation needs will center around mismatches between the selected intervention and target context (13–17). Where the purpose of adaptation is improving performance of an existing intervention, adaptation needs will center around identifying gaps between current and desired performance (16, 38). In some cases, the assessment phase may reveal a need to return to the intervention selection phase, either to complete key steps such as assessing core functions if they are not already known, or to fully select a new intervention if assessment reveals fundamental incompatibilities between the existing intervention and the current context that are unlikely to be rectified through adaptation.

In the preparation phase, stakeholders develop an adaptation plan and assemble an adapted intervention package, including adapted activities, an implementation plan, evaluation plan, and training materials (13–17). This adapted intervention package will use different intervention forms from the original intervention but should retain the same core functions (16, 40). In the final step of preparation, stakeholders build support for the adapted intervention package (e.g., recruiting local champions) (39), and recruit and train implementers (13–15, 17, 39).

In the implementation phase, stakeholders pilot test and refine the adapted intervention (14, 15, 17). During pilot testing, stakeholders assess implementation outcomes [e.g., acceptability, feasibility (80)], barriers and facilitators to adaptation, and any potential unintended consequences (e.g., instances where adaptation undermined core functions and reduced effectiveness). If pilot testing indicates substantial deficiencies, stakeholders may choose not to proceed to implementation at scale and instead return to earlier steps to iterate adaptation development (38). Otherwise, the adapted intervention package is refined, then implemented at scale.

In the sustainment phase, stakeholders evaluate the adaptation, following the evaluation plan identified in the preparation phase (15, 17). Then, assuming satisfactory performance, stakeholders work to systematize and sustain the adapted intervention through identifying opportunities for scale up and integrating the adapted intervention package into organizational structures and implementation systems (15).

Depending on the scale of the adaptation, stakeholder may need to dedicate proportionally more or less effort to specific steps and actions. For example, adaptations to tailor a behavior change messaging intervention to improve relevance to local behavioral drivers may not require securing formal permission from regulatory agencies but will require capacity building among implementers. This model is designed to be comprehensive for adaptations making major modifications. For smaller-scale adaptations, stakeholders may omit specific actions but should have appropriate justification for doing so.

#### Tools to Support Adaptation

In this section, we present tools that can support stakeholders in executing steps of the adaptation model. Table 4 provides a description of each tool, an example of its application, and indicates the adaptation model steps in which it can be applied. Table 3 describing the model steps also contains a column indicating applicable tools for each step.

We organized these tools into four groups based on their function: assessing context and conditions, understanding core functions and selecting adaptations, describing adaptations and implementation processes, and evaluating adaptations and implementation. We categorized each tool into only a single group based on its primary function, but some tools can serve multiple functions. For example, we included bottleneck analysis in the group of tools for assessing context and conditions, as bottleneck analysis can be used to determine barriers to achieving a particular health target. However, bottleneck analysis could also be used for describing implementation processes. We encourage stakeholders to apply these tools throughout the adaptation process wherever they are useful.

Tools for assessing context and conditions can be applied in the intervention selection and assessment phase for steps to understand the target population's needs and context. They may also be applied in the preparation, implementation, or evaluation phases to assess contextual barriers and facilitators to successful implementation. Tools for understanding core functions and selecting adaptations apply primarily in the intervention selection phase. These tools may also be useful in subsequent steps if stakeholders are adapting an existing intervention where documentation of the original intervention is poor or core functions are not well defined, and stakeholders require additional information to develop an adapted intervention plan.

Tools for describing adaptations and implementation processes can be used in the preparation stage to plan the adapted intervention package, in the implementation stage to understand and improve implementation, and in the sustainment phase to disseminate findings. These tools allow for more systematic and comprehensive description of the adaption and can also be used in combination with monitoring and evaluation data to explore barriers and drivers to success. Tools for evaluating adaptations and implementation are most applicable in the implementation and sustainment phases, to support steps for pilot testing and evaluation.

## Discussion

We developed an adaptation model tailored specifically to the context of WaSH programs. Adaptation is an important part of program cycle, facilitating implementation of evidence-based interventions while retaining their effectiveness. Our model is intended to improve outcomes by systematizing the adaptation process, as is consistent with literature showing that unsystematic adaptations have poorer outcomes (24). While a wealth of adaptation models and frameworks exist, they originate from a healthcare context, which differs substantially from the WaSH context. Limited tools are available to support adaptation outside the healthcare context. Our model addresses this evidence gap by specifically considering the needs of WaSH interventions implemented outside conventional health system channels.

Our model also contributes to the adaptation literature by synthesizing prior models and frameworks. Adaptation literature has seen proliferation of models and frameworks over the past two decades especially, but few attempts to synthesize them. New concepts (e.g., core functions) have been recognized but not yet integrated into existing models (13, 14). Proliferation of models and frameworks without comprehensive synthesis efforts can create challenges where concepts develop overlapping, conflicting, or ambiguous meanings (83). Our model combats this challenge by synthesizing a broad array of adaptation models and frameworks and aligning them with current concepts in adaptation literature.

Below, we discuss how our model updates and refines prior adaptation literature and the specific contextual needs of WaSH adaptation.

### Updates and Refinements to Prior Adaptation Models

Our model is grounded in previous adaptation models proposed by Movsisyan (15), Kirk (16), and Escoffery (17). Movsisyan et al. (15) propose 11 steps divided into four phases—exploration, preparation, implementation, and sustainment—and draw from prior literature proposing the same phases for *de novo* intervention delivery (84). Escoffery et al. (17) propose 11 similar steps, which are similarly ordered but not explicitly divided into phases.

These steps are well represented in examples of WaSH literature yielded by our systematic search. While studies did not explicitly reference these models or use their terminology, we found adaptation stakeholders commonly taking steps throughout all phases. For example, studies reported steps in the exploration phase to identify performance gaps and the need for adaptation (43, 67, 85), in the preparation phase to prepare the adapted intervention materials (19, 20, 48), in the implementation phase to pilot test and refine the adapted intervention (18, 44), and in the sustainment phase to evaluate the adapted intervention (56, 66). Most studies focused on the early stages of assessment and preparation to identify adaptation needs and propose solutions. We found fewer examples where proposed adaptations had been actually implanted and refined in pilot projects (44, 46, 56, 66, 67), and fewer still that had been evaluated in larger-scale trials and integrated into programming at-scale (49, 63).

Kirk et al.'s model (16) is aimed primarily at assessing whether adaptations are systematic and aligned with core functions of the original intervention. It provides constructs to describe adaptations, outlines steps to assess potential outcomes, and guides decision-making surrounding whether adaptations should proceed to implementation at scale based on their potential outcomes. It also makes an important contribution to the literature by incorporating steps to consider core functions as part of the adaptation process.

Our systematic literature search yielded no examples that referenced core functions, nor analogous concepts under a different name. We found two examples of WaSH adaptation taking a systematic approach to assessing potential outcomes, but this was done through empirical data collection on key pre-selected outcomes using continuous quality improvement framework (21, 86). Overall, the scope of potential outcomes considered by all WaSH studies was limited, typically only direct health impacts expected of the intervention (e.g., diarrheal disease prevalence) or intermediate outcomes such as functionality and use of WaSH infrastructure. We found few examples of studies anticipating or exploring other potential effects on other health or non-health impacts, despite evidence that WaSH intervention adaptation can have broad, unanticipated effects on mental, social, and economic wellbeing (12).

Our model retains analogous steps to those proposed by Movsisyan and Escoffery. We integrated Kirk's model by including specific actions for identifying core functions and potential outcomes throughout steps in the preparation and implementation phases. Throughout the model, we have also updated steps and actions to reflect the concept of core functions vs. forms. Models from Movsisyan and Escoffery contain actions for identifying and preserving important “core components” (an early variant of the concept of core functions). We have updated these to reflect current thinking on core functions and forms, as described by Kirk (16) and others (13, 22, 40).

Our model makes several important refinements to previous models. First, we proposed the addition of a distinct phase for intervention selection as the first phase in the adaptation process. In our model, we group the initial steps in both the Movsisyan and Escoffery models into a phase for intervention selection, and propose that some stakeholders may skip this first phase if they are adapting a preexisting intervention. When stakeholders begin with intervention selection, they are engaging in proactive adaptation (i.e., adaptations made in response to anticipated needs before they arise). When stakeholders skip the intervention selection phase and begin at assessment, they are engaging in reactive adaptation (i.e., adaptations made in response to unanticipated needs that arise during implementation (16, 31).

Reactive adaptation is the norm in WaSH and other sectors (24). Previous models include intervention selection steps as an essential part of the process, and provide little guidance for what to do in the case of reactive adaptation where an intervention is already in place. Our model better accounts for the realities of reactive intervention by separating these steps into a separate, optional phase. Second, we depict the adaptation process as a cycle. Movsisyan and Escoffery both recognize that some adaptation steps may require iteration but present their models as linear and offer little guidance for when iteration should occur. Kirk (16) and others (38) explicitly depict iterative adaptation design and refinement, but it is unclear where in the overall adaptation process this should occur. Our model puts this in context by depicting the assessment, implementation, and sustainment phases as a cycle that is initiated after intervention selection, which can be iterated for multiple rounds of adaptation and ongoing improvement throughout the adapted intervention delivery.

Third, we integrated actions to identify, engage, and build support among stakeholders throughout the model. This reflects the specific context of WaSH adaptation, where stakeholders are diverse and represent government, non-governmental organizations, and the private sector across multiple disciplines, as well as lay implementers and volunteers from within beneficiary communities. These stakeholders can have competing goals and objectives that require additional efforts to solicit and incorporate their feedback and build consensus, which is not reflected in non-WaSH specific adaptation models (1, 47, 61, 62, 87).

Fourth, we integrate a list of tools that can support data collection and decision-making throughout the adaptation process (Table 4). Prior models broadly describe the steps and actions required for adaptation but provide few tools to guide stakeholders explicitly through completing these actions.

### Specific Considerations for Adapting Interventions in the WaSH Context

Below, we discuss four specific contextual considerations for adapting WaSH interventions, which we derived from literature describing examples of WaSH adaptations presented in Table 1. They reflect commonly reported challenges, opportunities, and lessons learned in these studies. While not all these considerations are necessarily unique to WaSH, they introduce complexities into the adaptation process that warrant particular attention.

#### Multi-Component, Complex Interventions

WasH interventions are complex, often including both a hardware and a software component that are interdependent. Hardware interventions are the infrastructure or other physical goods that provide people access to WaSH, such as water taps and toilets used by individuals and large systems for water and sewerage treatment. Software interventions are the efforts to change WaSH practices by altering knowledge, attitudes, norms, and other behavioral drivers. Interventions can be either supply- or demand-side to increase availability of WaSH products for consumers or to increase consumer demand for those products, respectively. WaSH interventions may also target policy from the national to local levels.

Despite the interconnectedness of WaSH components, responsibilities for implementation, financing, monitoring, regulation are often fragmented across multiple stakeholders at various levels within government, non-governmental organizations, and the private sector. This fragmentation can create challenges with funding shortfalls, lack of leadership to coordinate and champion the original intervention and associated adaptation activities, and non-comprehensive monitoring efforts (47, 61, 87). Furthermore, poorly aligned objectives and insufficient coordination and communication between stakeholders can lead to competing and conflicting adaptations (1, 62). Additional efforts may be needed to generate buy-in from stakeholders in the preparation phase, compared to interventions in other sectors with fewer or less diverse stakeholders (53). Evaluation frameworks and multiple criteria decision analysis may be useful to describe intervention and adaptation goals more comprehensively and to facilitate compromises between differing stakeholder priorities.

#### Identifying Core Functions and the “Performance Envelope”

Many WaSH programs have been critiqued for lacking well-defined core functions (1, 87, 88). Disentangling core functions of multiple simultaneous interventions at different levels (e.g., policy-level subsidies and household-level behavior change messaging for sanitation) can be particularly challenging but is important for effective adaptation (13). For interventions where core functions are not already well defined, stakeholders may need to allocate time and resources to identify them. Methods for identifying core functions are beyond the scope of this paper but have been described elsewhere (22).

Recent studies have suggested that WaSH interventions have a “performance envelope” (i.e., a limited range of settings in which the contextual conditions align with the core functions of the intervention) (89, 90). For example, one study of a participatory urban sanitation intervention in which community engagement was critical to the theory of change concluded that the intervention should only be selected for adaptation in areas where communities show “willingness to participate in planning, training, and operation and maintenance” (44). Similarly, the specific pathogens and transmission pathways contributing to the burden of disease will vary by context, such that interventions to control the predominant transmission pathway in one setting may not produce comparable effects in another setting (1). Tools such as behavior change theories and intervention mapping can help identify incompatibilities between core functions and the intended context.

#### Decentralized Programming and Lay Implementers

Adaptation models developed for medical and public health settings typically assume that implementers and beneficiary populations are distinct groups, and that implementers are trained professionals. However, in WaSH these assumptions may not hold. Some WaSH programs are decentralized, with governance, planning, and implementation being conducted at the village or sub-regional level (47). Programs may emphasize recruitment of community volunteers and other lay individuals to organize and champion grassroots implementation efforts. Furthermore, WaSH programs typically have a distinct health goal but are often implemented and managed by non-health ministries, organizations, and other entities. Stakeholder mapping can be used to explore the diversity of relevant stakeholders and map their priorities and capabilities (42, 44).

Decentralization and lay implementers require specific consideration for adaption in WaSH compared to interventions in other sectors. Lay implementers have deep knowledge of the local context that makes them highly effective at designing contextually appropriate adaptations (91), and various studies have emphasized the importance of engaging community members and local-level implementers in decision-making processes (44, 45, 60). However, studies have also found that institutional support structures may be lacking in highly decentralized programs, and low access to information, funding, and support structures can hinder adaptation (62).

In response, adaptation in WaSH will likely require additional effort, time, and resources to fully engage and leverage the knowledge and skills of local level implementers and community members. WaSH programs may benefit from parallel efforts to build the skills of local-level implementers to autonomously engage in formal data collection and research and program evaluation efforts (12). In some cases, modifying or simplifying tools to make them more user-friendly may be necessary to improve engagement of community members and lay implementers and to improve overall program outcomes (56).

#### Inclusive Interventions

WaSH programs often target entire communities and aim to reach universal coverage. Universal coverage targets are informed by evidence that community-wide coverage is necessary to achieve “herd protection,” where exposure to fecal pathogens is reduced not only within the home but also in the broader community environment (92). However, targeting an entire community's population can raise challenges to ensure that adapted interventions are accessible and appropriate for all. In some cases, multiple adaptations may be necessary to develop different tailored versions of the intervention that separately address different demographics within a community, as individuals' specific WaSH needs and preferences will vary based on factors such as age, gender, and ability (48). Efforts to engage community members throughout the adaptation process, especially vulnerable and marginalized populations, is important for ensuring that adapted interventions are inclusive for all.

### Strengths, Limitations, and Next Steps

The purpose of study was to identify the steps required for successful adaptation of WaSH interventions and to identify tools to guide these steps. Our scoping review methods were designed to explore the breadth of available literature and synthesize key principles and illustrative examples, rather than to systematically identify all cases. We identified broad categories of tools to support data collection and decision-making in adaptation, and the specific examples included under each category are not intended to be exhaustive. It is also likely that sectors outside of WaSH use different tools that may also aid WaSH adaptation, which this search methodology was not designed to capture. Future systematic reviews to identify tools more narrowly but deeply within each category could aid future adaptation research and practice.

We prioritized our review for adaptation literature from the past 5 years, and we restricted our search to WaSH studies published from 2000 onwards. These search periods reflect the newness of adaptation as an area of systematic study. Adaptation as a subfield of implementation science has undergone substantial evolution in recent years [for an in-depth discussion, see, e.g., (34, 36)]. Concepts from earlier literature been revised in favor of the more current concepts we present here, and we therefore did not prioritize this early literature in our search. Similarly, adaption has been the norm in WaSH for decades, but literature systematically describing the process using concepts from adaptation literature (or early analogous ideas) prior to 2000 is unlikely.

We found few instances where models, frameworks, and other tools from adaptation literature were applied in studies of WaSH adaptation, even within recent literature. The studies of WaSH adaptation that we reviewed often developed their own tools as part of the study, rather than drawing on existing tools in adaptation literature. Yet in many cases, these tools were purpose-built and had low generalizability beyond the original study context. Tools in adaptation literature were more generalizable across a broad range of contexts. While they have not yet been widely applied in WaSH, they offer the potential to improve the efficiency and effectiveness of adaptation by reducing the burden to develop new tools for every context and improving the ability to compare and apply learnings across settings.

Under-utilization of tools from adaptation literature in WaSH likely reflects in part the newness of adaptation as a discipline, and we expect that application of adaptation tools in WaSH will increase over time. We expect that our model may need revision as adaptation literature disseminates more broadly into WaSH research and practice, and more evidence becomes available.

## Conclusions

Adaptation of WaSH interventions is common but does not always follow a systematic process. Unsystematic adaptation can lead to sub-optimal or even detrimental outcomes, such as decreased effectiveness if adaptations undermine core functions (11) or even harm if modifications are made without considering the range of possible detrimental effects on physical, mental, and social wellbeing (12). Adaptation literature offers a variety of tools to guide stakeholders through the process of designing, implementing, and evaluating adaptations to facilitate implementation while retaining effectiveness. However, these tools have rarely been applied in WaSH, and they were designed predominantly for interventions in a healthcare context, which differs substantially from the WaSH context in terms of the diversity of health and non-health actors involved in implementation and regulation.

We developed a model and framework of tools to assist adaptation tailored specifically to the WaSH context. Our model is underpinned primarily by evidence of household-level WaSH interventions, though we expect it could be successfully applied in institutional contexts as well (e.g., healthcare, schools) settings as well. We anticipate that application of this model will improve the effectiveness of adapted WaSH interventions by systematizing the adaptation process. Our model advances implementation science literature by proposing steps for adapting interventions that are implemented outside the healthcare sector by diverse lay and non-health stakeholders, and by specifically identifying tools that can support data collection and decision-making for more systematic adaptation processes and improved program outcomes.

## Author Contributions

DA, JB, and MF: conceptualization. DA: formal analysis and writing—original draft. DA, SB, JB, and MF: writing—review and editing. All authors read and approved the final manuscript.

## Funding

DA was supported by grants from the University of North Carolina Royster Society of Fellows and from the National Institute of Environmental Health Sciences (T32ES007018). The funders had no role in the design of the study and collection, analysis, and interpretation of data and in writing the manuscript, or decision to submit for publication.

## Conflict of Interest

The authors declare that the research was conducted in the absence of any commercial or financial relationships that could be construed as a potential conflict of interest.

## Publisher's Note

All claims expressed in this article are solely those of the authors and do not necessarily represent those of their affiliated organizations, or those of the publisher, the editors and the reviewers. Any product that may be evaluated in this article, or claim that may be made by its manufacturer, is not guaranteed or endorsed by the publisher.
