# Supplementary material for: Adaptation of Water, Sanitation, and Hygiene Interventions: A Model and Scoping Review of Key Concepts and Tools
Source: Front Health Serv. 2022 May 9;2:896234. doi: 10.3389/frhs.2022.896234 (PMC10012759; doi:10.3389/frhs.2022.896234)
Supplement: Supplementary file 1 [file Data_Sheet_1.docx]

Supplemental File 1

Search terms and methods

**Overview**

**Databases queried**: PubMed, SCOPUS, Web of Science

**Search date:** 21 June 2021

**Search terms**

Each database was queried with the following two search strings:

Search string 1

(adaptation OR adaptations) AND (model OR theory OR theories OR framework OR tool OR toolkit) AND (“drinking water” OR sanitation OR hygiene OR handwashing)

Search string 2

(adaptation OR adaptations) AND (water AND sanitation AND hygiene)

Fields searched were titles, abstracts, and keywords. Searches were restricted to studies published 1 January 2000 or later

**Search strings by database**

**PubMed**

Search date: 21 June 2021

Fields searched: title/abstract

Restrictions: studies published on or after 1 January 2000

Search string 1

(adaptation[Title/Abstract] OR adaptations [Title/Abstract]) AND (model[Title/Abstract] OR theory[Title/Abstract] OR theories[Title/Abstract] OR framework[Title/Abstract] OR tool[Title/Abstract] OR toolkit[Title/Abstract]) AND (“drinking water”[Title/Abstract] OR sanitation[Title/Abstract] OR hygiene[Title/Abstract] OR handwashing) (adaptation[Title/Abstract] OR adaptations)[Title/Abstract] AND (water[Title/Abstract] AND sanitation[Title/Abstract] AND hygiene[Title/Abstract])

Search string 2

(adaptation[Title/Abstract] OR adaptations[Title/Abstract]) AND (water[Title/Abstract] AND sanitation[Title/Abstract] AND hygiene[Title/Abstract])

**SCOPUS**

Search date: 21 June 2021

Fields searched: article title, abstract, keywords

Restrictions: studies published on or after 1 January 2000

Search string 1

TITLE-ABS-KEY((adaptation OR adaptations) AND (model OR theory OR theories OR framework OR tool OR toolkit) AND (“drinking water” OR sanitation OR hygiene OR handwashing))

Search string 2

TITLE-ABS-KEY((adaptation OR adaptations) AND (water AND sanitation AND hygiene))

**Web of Science**

Search date: 21 June 2021

Fields searched: Topic (title, abstracts, keywords)

Restrictions: studies published on or after 1 January 2000

Search string 1

TS=((adaptation OR adaptations) AND (model OR theory OR theories OR framework OR tool OR toolkit) AND (“drinking water” OR sanitation OR hygiene OR handwashing))

Search string 2

TS=((adaptation OR adaptations) AND (water AND sanitation AND hygiene))

**PRISMA flow diagram of search steps**

Studies removed *before screening*:

Duplicates removed (n = 752)

Studies identified (n = 2,401)

**Identification**

Studies not retrieved

- Full text not available (n = 4)

Studies screened - full text (n = 94)

Studies sought for retrieval (n = 98)

Studies screened - title/abstract (n = 1,649)

Studies excluded (n = 1,551)

**Screening**

Studies excluded:

- Non-WaSH intervention (n=42)
- WaSH intervention but no description of adaptation (n=20)
- WaSH intervention in ineligible setting (n=2)

Studies included in review (n = 30)

**Included**
