# Supplementary material for: Adaptation of Water, Sanitation, and Hygiene Interventions: A Model and Scoping Review of Key Concepts and Tools
Source: Front Health Serv. 2022 May 9;2:896234. doi: 10.3389/frhs.2022.896234 (PMC10012759; doi:10.3389/frhs.2022.896234)
Supplement: Supplementary file 2 [file Data_Sheet_2.DOCX]

Supplemental File 2

Details of WaSH interventions in included studies

Integrating mobile technologies

Murthy et al. 2018

**Setting**: global but predominantly LMICs

**Target population**: mixed, community and institutional

**Intervention summary**: not an intervention but a case study of nine different mobile data collection tools for monitoring and evaluating WaSH interventions

Urban household WaSH

Medilanski et al. 2007

**Setting**: peri-urban China

**Target population**: households

**Intervention summary**: urine diverting toilets designed to cope with water scarcity and population growth in urban contexts

Tidwell et al. 2019.

**Setting**: urban Zambia

**Target population**: groups of households in urban compounds

**Intervention summary**: quality improvement intervention to improve funcationality, cleaning, and moverall maintenance by tenants and landlords for shared sanitation in urban compounds

Lako et al. 2015.

**Setting**: urban Cameroon

**Target population**: households

**Intervention summary**: urine diverting toilets designed to cope with flooding

Sigel et al. 2014.

**Setting**: peri-urban Mongolia

**Target population**: households

**Intervention summary**: community-led total sanitation approach originally developed in rural areas applied in an urban setting

Putri et al. 2020.

**Setting**: urban Indonesia

**Target population**: households

**Intervention summary**: case study of households in informal settlements, challenges they face, and adaptation strategies used to fullfill water and sanitation needs

Rural household WaSH

Ogunjobi et al. 2013.

**Setting**: rural Nigeria

**Target population**: households

**Intervention summary**: reconstruction and rehabilitation of household latrines following a community-led total sanitation intervention

Nordhauser et al. 2020.

**Setting**: rural Domican Republic

**Target population**: households

**Intervention summary**: community-health clubs to promote adoption of healthy WaSH behaviors for handwashing, personal hygiene, sanitation, and environmental management

Manjang et al. 2018.

**Setting**: rural Gambia

**Target population**: households

**Intervention summary**: community-level activities to promote household-level hygiene behaviors, adapted from interventions originally developed in India and Nepal

Jimenez et al. 2017.

**Setting**: eight countries in West Africa

**Target population**: mixed, predominantly rural households

**Intervention summary**: not an intervention but a framework designed to assess predictors of sustainability and continuous adaptation and improvement over time in water and sanitation services

Kamban et al. 2013.

**Setting**: rural Mali, Ghana, and Niger

**Target population**: households

**Intervention summary**: adapting existing household WaSH interventions to be more inclusive and meet the needs of persons living with disabilities

De Shay et al. 2020.

**Setting**: rural India

**Target population**: households

**Intervention summary**: community-level activities to promote household-level latrine use and safe disposal of child feces

Mehta et al. 2020.

**Setting**: rural India

**Target population**: households

**Intervention summary**: economic self-help and micro-credit groups adapted to add healh education modules to promote safe WaSH and nutrition behaviors

Municipal utilities or community-based water systems

Tscheikner-Gratl et al. 2014.

**Setting**: urban Austria

**Target population**: municipal utilities

**Intervention summary**: rehabilitation of an urban sewer network to respond to climate change and growing urban population

Curk et al. 2020.

**Setting**: urban Slovenia

**Target population**: municipal utilities

**Intervention summary**: adaptating municipal water supplies to respond to climate change and changing land use patterns

Barrington et al. 2013

**Setting**: rural Nepal

**Target population**: community-managed water systems

**Intervention summary**: integrating water safety plannign approach into communtiy-managed water systems

Climate change adaptation

Van Engelenburg et al. 2019.

**Setting**: global

**Target population**: mixed, household and institutional

**Intervention summary**: not an intervention but a framework to assess sustainability and adaptation to climate change and urbanization in drinking water sources and utilities

Hasse et al. 2016.

**Setting**: urban Germany

**Target population**: municipal utilities

**Intervention summary**: climate change adaptation among water utilities through steering groups, roadmapping, and other urban planning tools

Garnier. 2019.

**Setting**: urban European countries

**Target population**: municipal utilities

**Intervention summary**: case study of possible climate change adaptation measures among water utilities in the European context

Clark et al. 2011.

**Setting**: global, predominantly the United States and other high-income settings

**Target population**: municipal utilities

**Intervention summary**: not an intervention but a framework for a three-step approach for assessing climate change impacts and designing solutions in water utilities

Ojomo et al. 2016.

**Setting**: coastal areas of Vietnam and Philippines

**Target population**: mixed, household and institutional

**Intervention summary**: not an intervention but a framework to describe barriers to climate change adaptation in coastal communities

Mukheibir et al. 2017.

**Setting**: rural Kiribati

**Target population**: household and community-managed water systems

**Intervention summary**: community-level workshops to build capacity of community members for climate change adaptation for drinking water supplies

Kohlitz et al. 2019.

**Setting**: global

**Target population**: community-managed water systems

**Intervention summary**: not an intervention but a framework of factors supporting climate change adaptation in community-managed water systems

Chan et al. 2020.

**Setting**: rural Solomon Islands

**Target population**: community-managed water and sanitation systems

**Intervention summary**: not an intervention but a framework of factors supporting climate change adaptation in community-managed water and sanitation systems

Bollinger et al. 2014.

**Setting**: global

**Target population**: mixed, WaSH infrastructure systems in various settings

**Intervention summary**: not an intervention but a framework of factors supporting climate change adaptation in WaSH infrastructure systems

Alhassan et al. 2017.

**Setting**: rural Ghana

**Target population**: municipal WaSH planning committees

**Intervention summary**: not an intervention but case study of climate change adaptationm mainstreaming and barriers in WaSH planning at the municipal level

Public washrooms

Dawson et al. 2017.

**Setting**: not reported

**Target population**: mixed, primarily institutional (e.g., healthcare, food preparation settings)

**Intervention summary**: adpated hand hygiene technology for automated sinks

Hand hygiene in healthcare settings

Salmon et al. 2015

**Setting**: LMICs

**Target population**: healthcare facilities

**Intervention summary**: World Health Organization’s “My five moments for hand hygiene” intervention adapted for overcrowded settings in resource-limited health systems

Muller et al. 2020.

**Setting**: peri-urban Guinea

**Target population**: healthcare facilities

**Intervention summary**: assessing context-specfic barriers to hand hygiene and integrating and adapting components of World Health Organization’s hand hygiene promotion stategy in response

Schools

Lang et al. 2012.

**Setting**: rural Ghana

**Target population**: schools

**Intervention summary**: hand hygiene intervention to provide health education and tools for hand hygiene to primary school children
